# Supplementary material for: XOmiVAE: an interpretable deep learning model for cancer classification using high-dimensional omics data
Source: Brief Bioinform. 2021 Aug 17;22(6):bbab315. doi: 10.1093/bib/bbab315 (PMC8575033; doi:10.1093/bib/bbab315)
Supplement: supplementary_bbab315 [file supplementary_bbab315.pdf]

# XOmiVAE: an interpretable deep learning model for cancer classification using high-dimensional omics data – Supplementary information

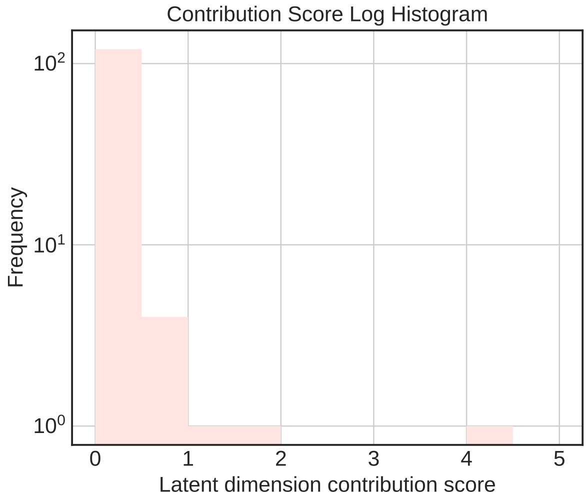


Supplementary Figure 1: Log histogram of the omics latent dimension contribution scores for LUAD prediction

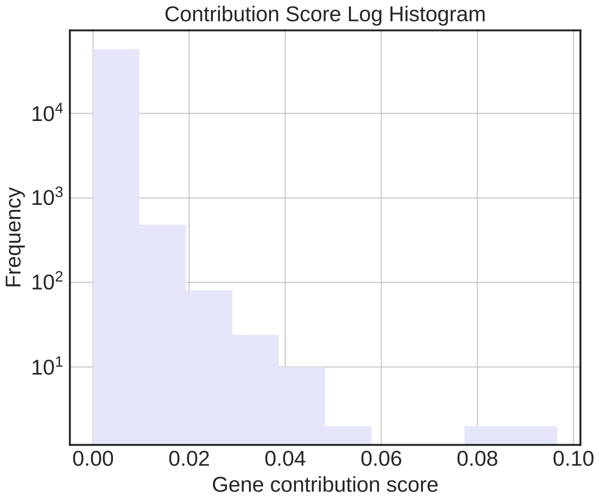


Supplementary Figure 2: Log histogram of the gene contribution scores for LUAD prediction

| Term ID | Term Name | Padj |
|---|---|---|
| GO:0030198 | Extracellular matrix organisation | $6.450 \times 10^{-11}$ |
| GO:0043062 | Extracellular structure organisation | $6.839 \times 10^{-11}$ |
| GO:0005615 | Extracellular space | $4.048 \times 10^{-12}$ |
| GO:0005576 | Extracellular region | $3.436 \times 10^{-16}$ |
| GO:0031012 | Extracellular matrix | $3.295 \times 10^{-18}$ |

Supplementary Table 1: A list of GO terms that are significantly overrepresented in the top 100 genes with highest contribution scores for BRCA. The GO term definitions were obtained from EMBL-EBI.

| Gene name | Contribution value | DEGs? |
|---|---|---|
| LYVE1 | 0.08802652 | True |
| COMP | 0.07973582 | True |
| SCARA5 | 0.07240529 | True |
| SCGB2A2 | 0.07231501 | False |
| LPL | 0.07080267 | True |
| PAMR1 | 0.07024122 | True |
| MMP11 | 0.06332003 | True |
| FABP4 | 0.06088565 | True |
| CFD | 0.06078836 | False |
| CCL21 | 0.06023567 | False |
| COL10A1 | 0.05834333 | True |
| MYOC | 0.05211987 | True |
| LTF | 0.05140609 | False |
| HBB | 0.05027783 | True |
| PCOLCE2 | 0.04830263 | True |
| SCGB1D2 | 0.0469837 | False |
| AC005152.3 | 0.04682791 | False |
| HSPB6 | 0.04676779 | True |
| CES1 | 0.04491388 | True |
| STAC2 | 0.04475317 | False |
| RBP4 | 0.04437602 | True |

| | | |
|---|---|---|
| TMEM132C | 0.04436617 | True |
| GSTA1 | 0.04426087 | False |
| KRT81 | 0.04423712 | False |
| WISP1 | 0.04395866 | True |
| HSPB7 | 0.04390443 | True |
| LEP | 0.04379686 | True |
| CIDEA | 0.04358062 | True |
| IGFBP6 | 0.04235139 | True |
| MMP3 | 0.04183716 | False |
| RP11-417E7.2 | 0.04122007 | False |
| CXCL2 | 0.04058203 | True |
| NRN1 | 0.04055257 | False |
| NPY2R | 0.04038753 | True |
| HBA2 | 0.03953991 | True |
| DEGS2 | 0.0390564 | False |
| MMRN1 | 0.03803924 | True |
| MFAP4 | 0.03684358 | False |
| PDLIM3 | 0.03567977 | False |
| DEFB1 | 0.03564727 | False |
| MUCL1 | 0.03556658 | False |
| COX6CP1 | 0.03456202 | False |
| MYEOV | 0.03400248 | False |
| ALDOC | 0.03336278 | False |
| CTHRC1 | 0.03331805 | False |
| RP11-92A5.2 | 0.031692 | False |
| MYH11 | 0.03150566 | True |
| RSPO3 | 0.03146641 | False |
| CYP4Z1 | 0.03132155 | False |
| AARD | 0.03101798 | False |
| ADIPOQ | 0.03096839 | True |
| EN1 | 0.03067887 | False |
| IRX1 | 0.03019223 | False |
| CNN1 | 0.0301683 | True |
| CLEC3B | 0.02989807 | True |
| CHRDL1 | 0.02956831 | True |
| RGS2 | 0.02956212 | False |
| ADH1C | 0.02949688 | True |
| PENK | 0.02940149 | False |
| NNAT | 0.02905503 | True |
| TPSP2 | 0.02874485 | False |
| FAM162B | 0.02874344 | False |
| FMO2 | 0.02873539 | True |
| NKAIN1 | 0.02868808 | True |
| DES | 0.02867935 | False |
| ANGPTL7 | 0.02847075 | True |
| PI16 | 0.02837563 | True |
| TBX15 | 0.02816985 | False |
| AC097713.3 | 0.027969 | False |
| FOS | 0.02796617 | False |
| SAMD5 | 0.02795375 | False |
| RP11-736K20.4 | 0.02790227 | False |
| UBE2Q2P6 | 0.02789169 | False |
| CBX4 | 0.02774911 | False |
| KLF4 | 0.02768696 | False |
| MATN3 | 0.02767189 | False |
| MAMDC2 | 0.02758051 | True |
| ALDH1A1 | 0.02732193 | False |
| HOXA9 | 0.02702148 | False |
| PPARG | 0.026999 | True |
| HRCT1 | 0.02693174 | False |
| MISP | 0.026907 | False |
| HOXC13 | 0.02684432 | False |
| MAOA | 0.02663056 | True |
| SERPINA5 | 0.02651533 | False |
| CAV1 | 0.02647178 | True |
| RP11-211G23.2 | 0.02636731 | False |
| SLC13A2 | 0.02623627 | False |
| CPB1 | 0.02623 | True |
| SLC29A4 | 0.02618126 | False |
| TMEM37 | 0.02603649 | False |
| PLAC9 | 0.02602616 | False |
| CTXN1 | 0.02567291 | False |
| BTNL9 | 0.0255975 | True |
| RP11-736K20.5 | 0.02552646 | False |
| DNAJC12 | 0.02540714 | False |
| FOSB | 0.0251986 | True |
| IGHA2 | 0.02517499 | False |

Supplementary Table 2: The top 100 contribution genes for BRCA prediction compared to the differentially expressed genes (DEGs).

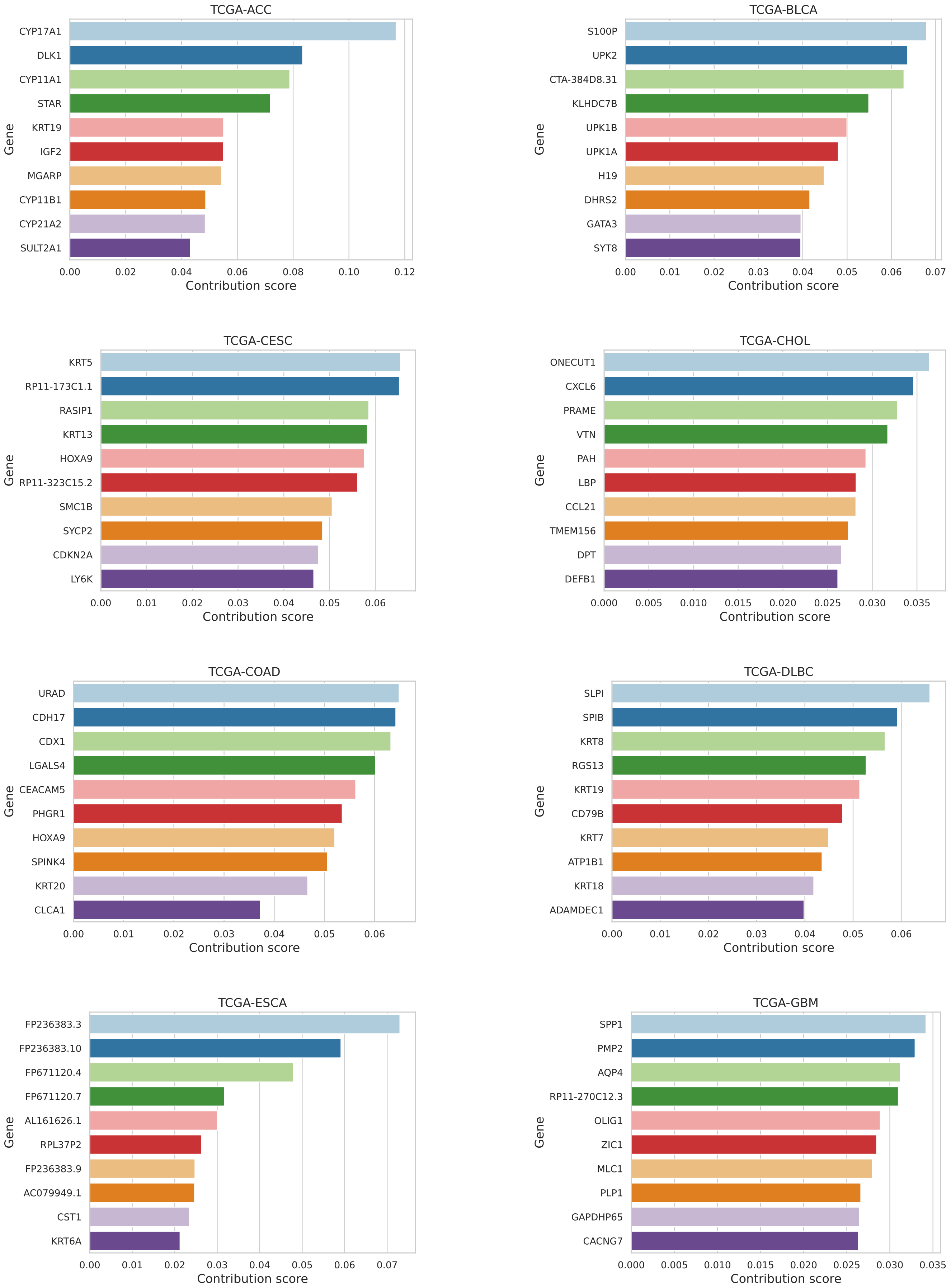


Supplementary Figure 3: The top 10 genes for TCGA tumour types ACC, BLCA, CESC, CHOL, COAD, DLBC, ESCA and GBM.

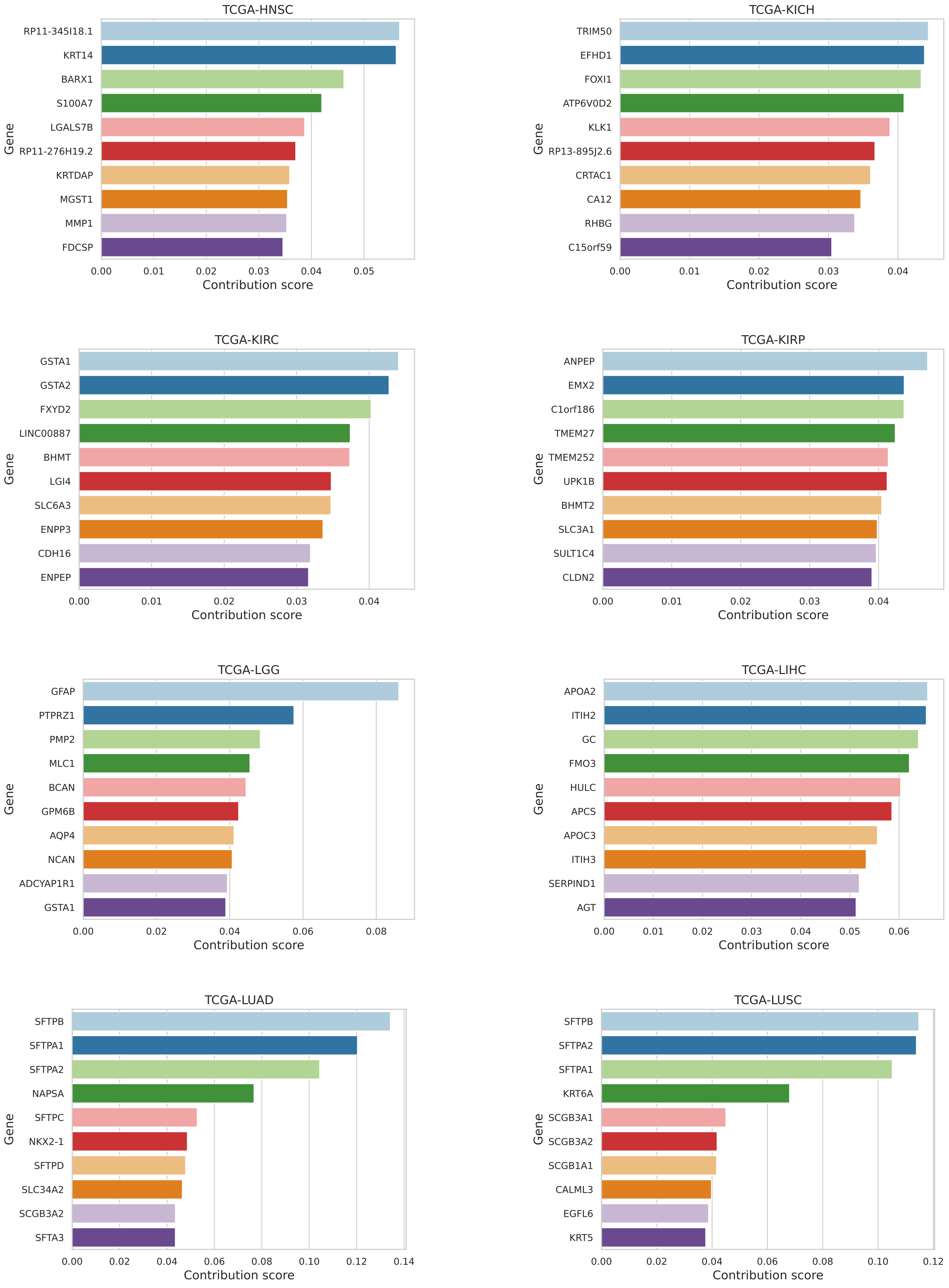


Supplementary Figure 4: The top 10 genes for TCGA tumour types HNSC, KICH, KIRC, KIRP, LGG, LIHC, LUAD and LUSC.

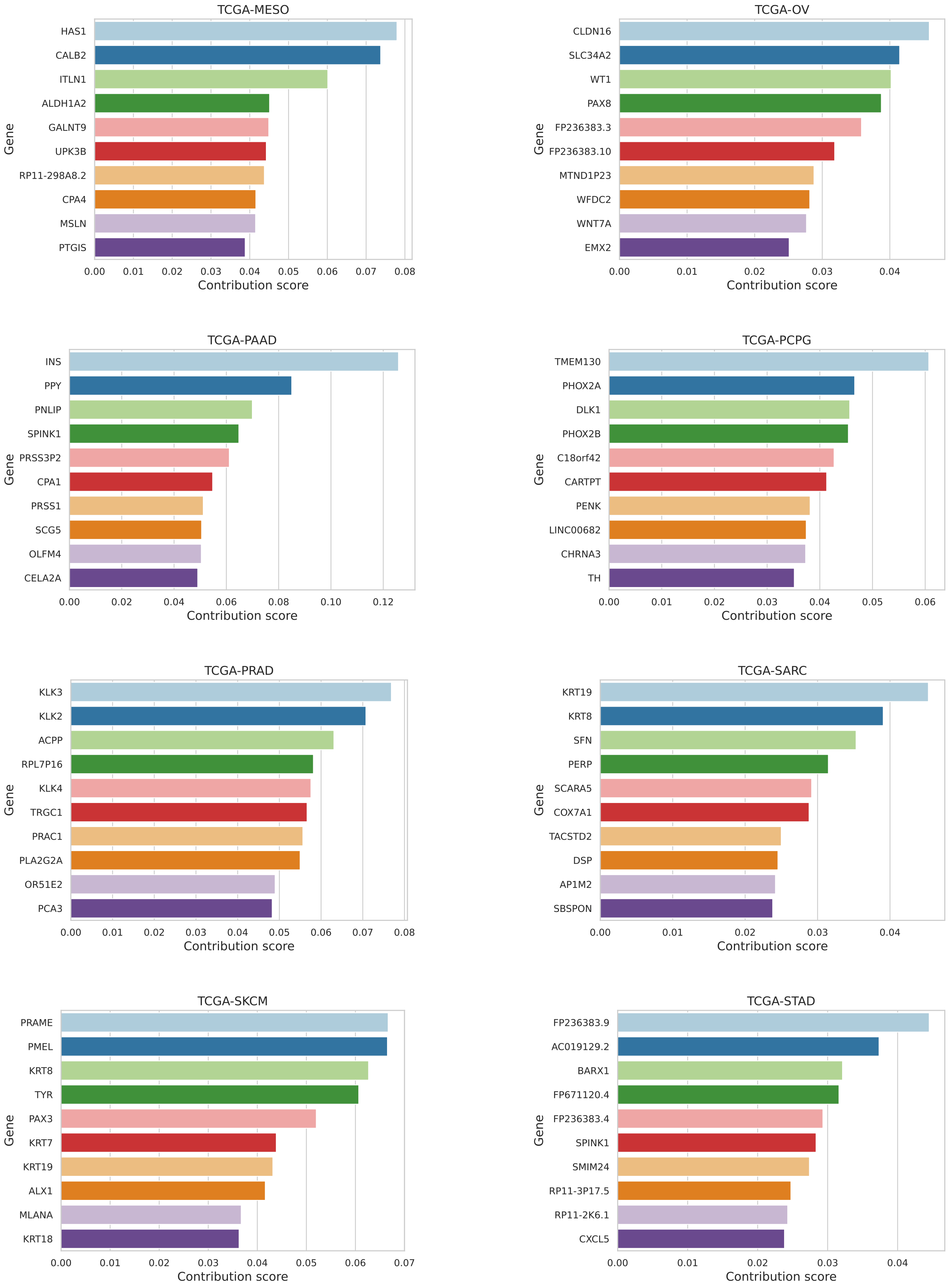


Supplementary Figure 5: The top 10 genes for TCGA tumour types MESO, OV, PAAD, PCPG, PRAD, SARD, SKCM and STAD.

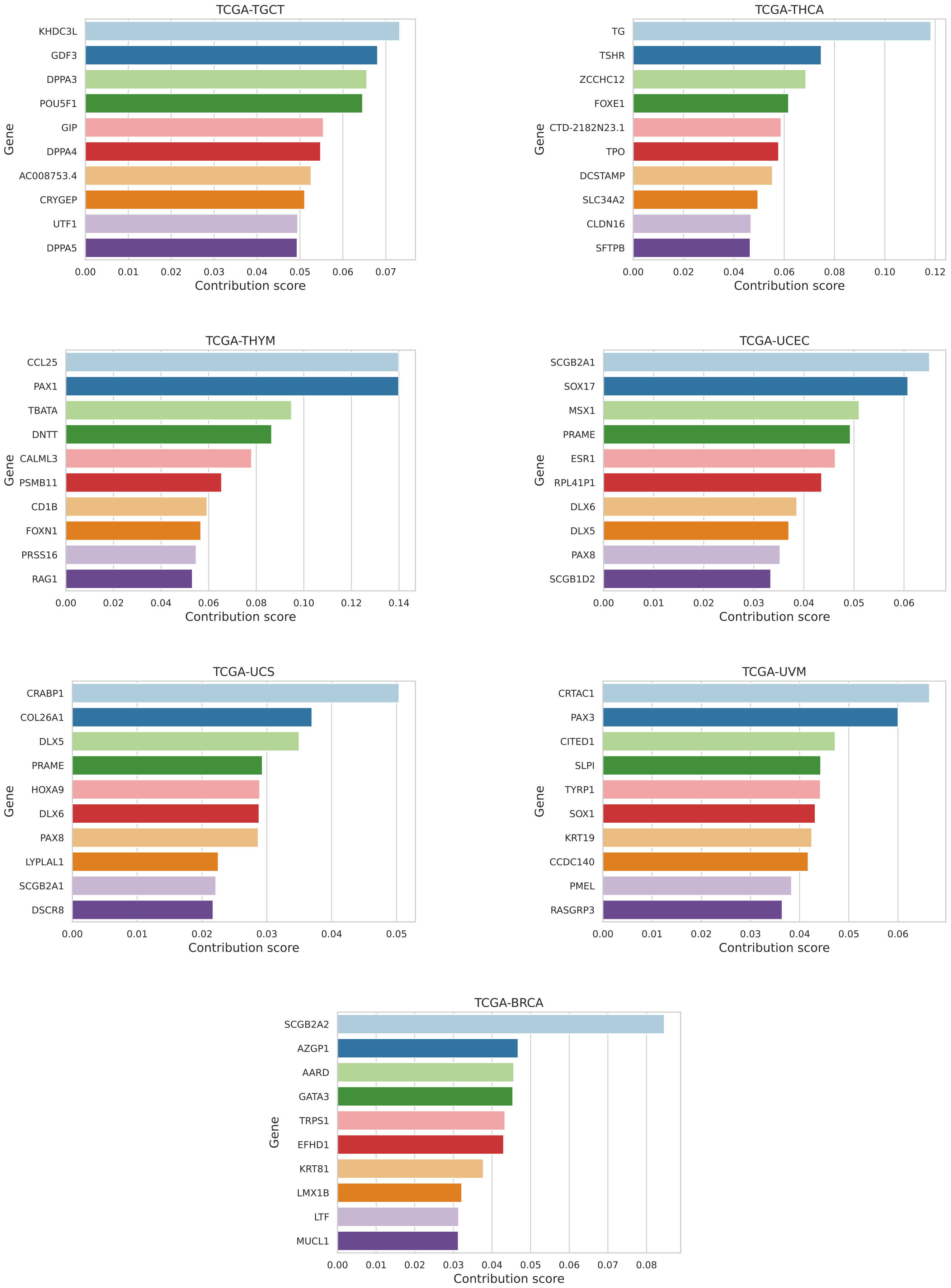


Supplementary Figure 6: The top 10 genes for TCGA tumour types TGCT, THCA, THYM, UCEC, UCS, UVM and BRCA.

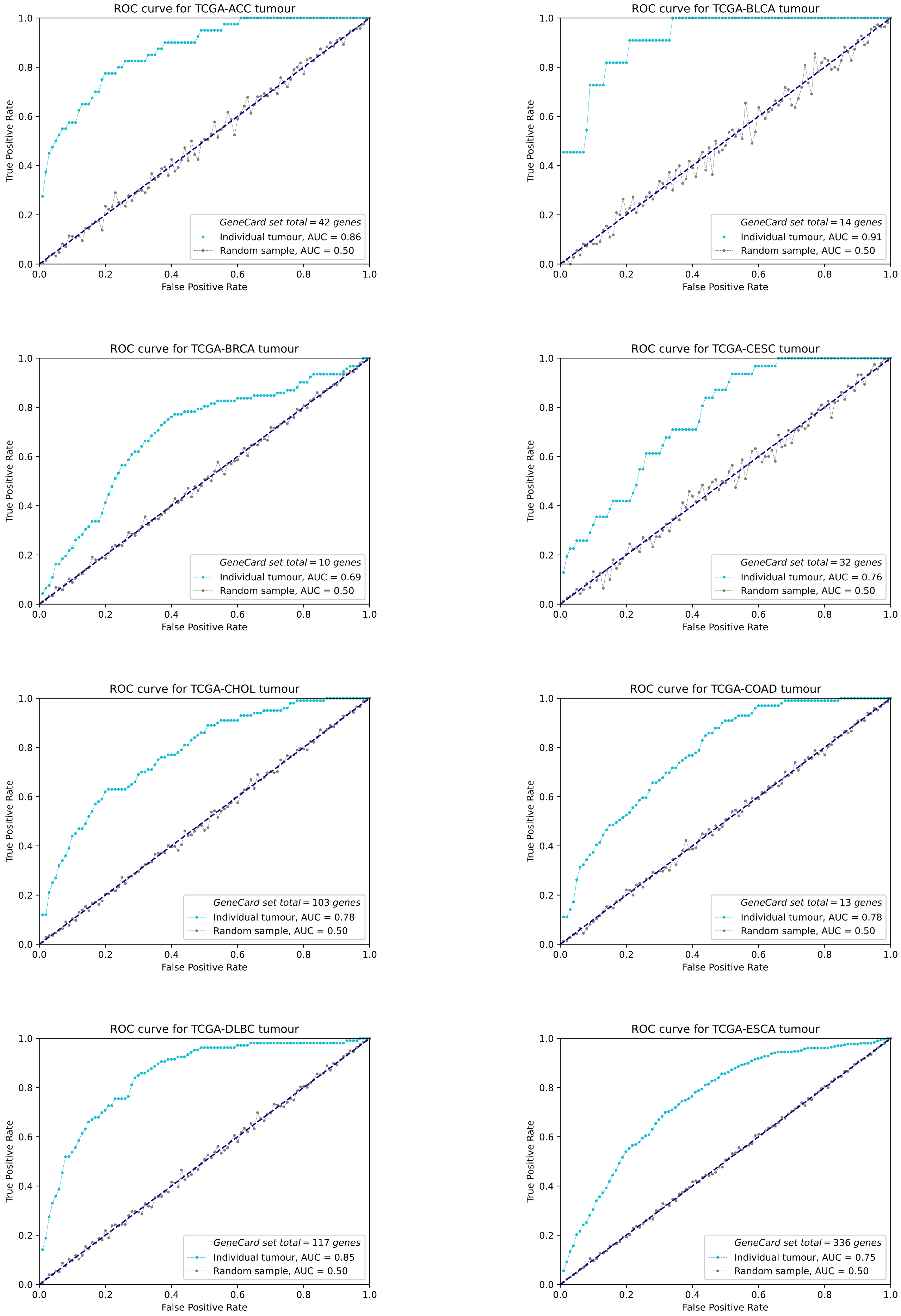


Supplementary Figure 7: AUC-ROC curves for the TCGA tumour most important genes (top SHAP genes) and random samples of genes against the GeneSet gene list for the tumour type. 100 thresholds were used, spaced evenly from 0 to 58,043 (total number of genes). A high AUC score indicates a higher number of genes that matched with the relevant disease genes in the GeneSet database.

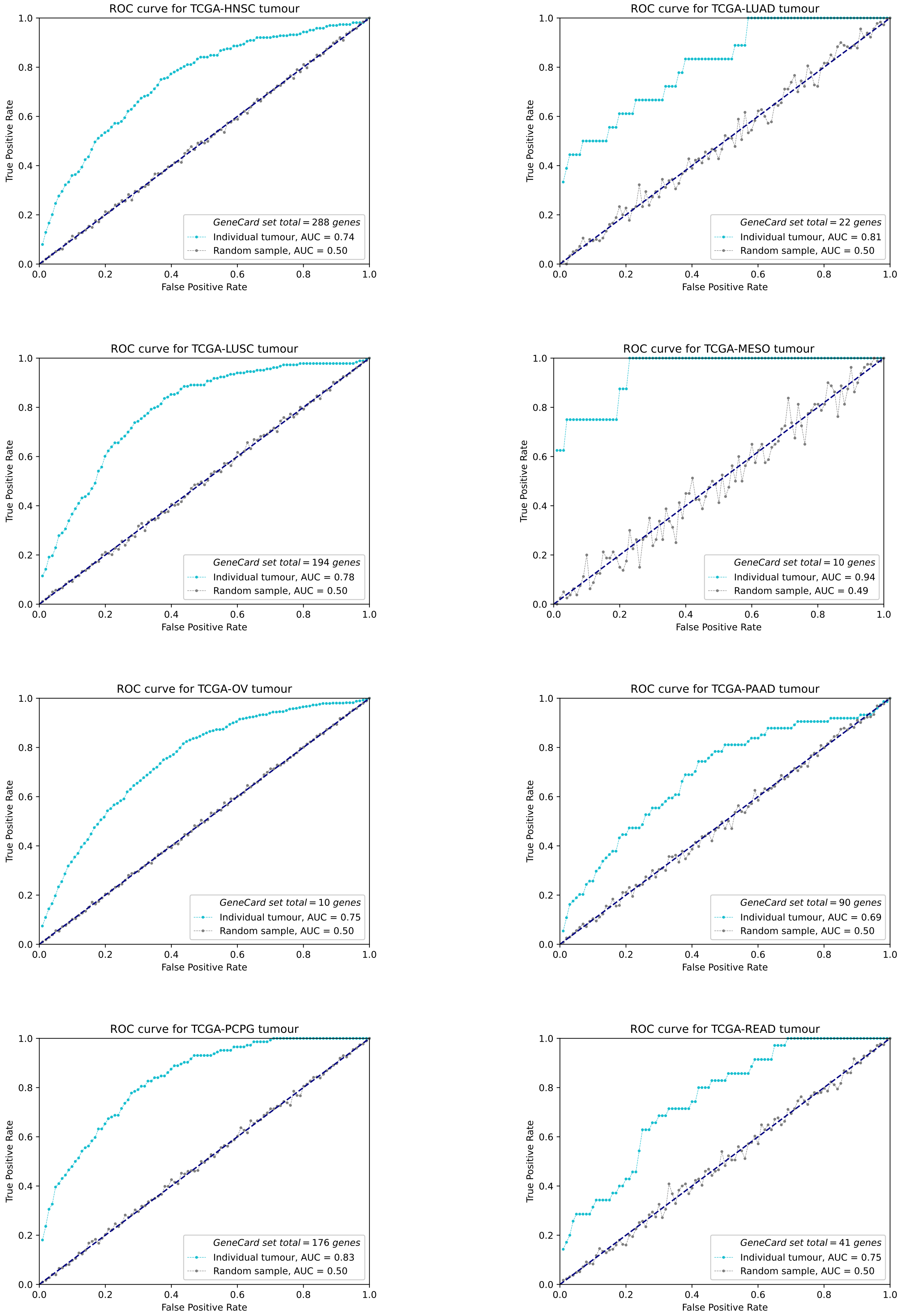


Supplementary Figure 8: AUC-ROC curves for the TCGA tumour most important genes (top SHAP genes) and random samples of genes against the GeneSet gene list for the tumour type. 100 thresholds were used, spaced evenly from 0 to 58,043 (total number of genes). A high AUC score indicates a higher number of genes that matched with the relevant disease genes in the GeneSet database.

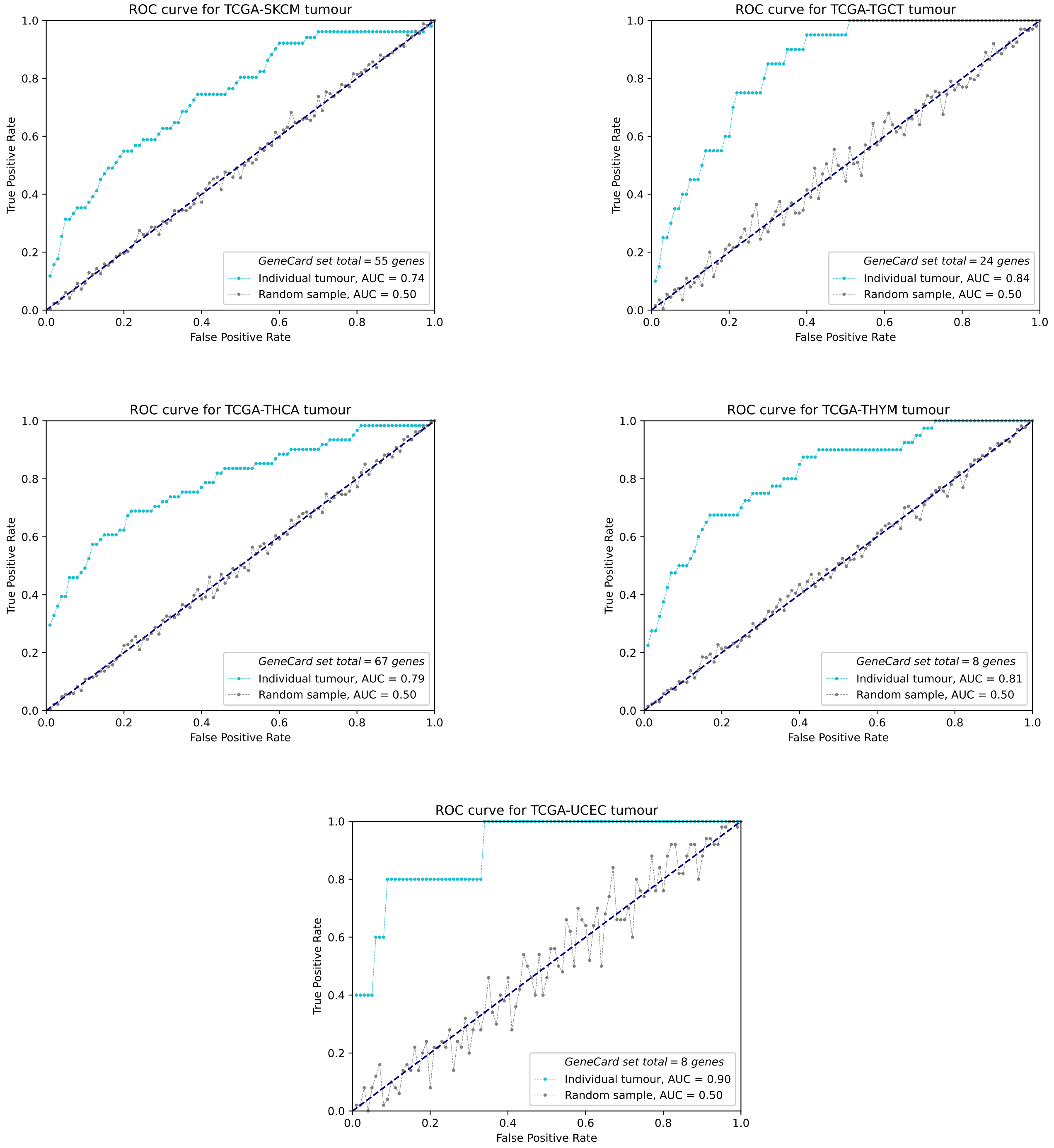


Supplementary Figure 9: AUC-ROC curves for the TCGA tumour most important genes (top SHAP genes) and random samples of genes against the GeneSet gene list for the tumour type. 100 thresholds were used, spaced evenly from 0 to 58,043 (total number of genes). A high AUC score indicates a higher number of genes that matched with the relevant disease genes in the GeneSet database.

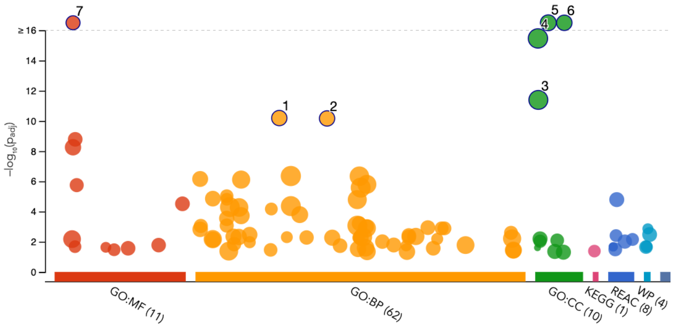


| ID | Source | Term ID | Term Name | $p_{adj}$ (query_1) |
|---|---|---|---|---|
| 1 | GO:BP | GO:0030198 | extracellular matrix organization | $6.450\times10^{-11}$ |
| 2 | GO:BP | GO:0043062 | extracellular structure organization | $6.839\times10^{-11}$ |
| 3 | GO:CC | GO:0005615 | extracellular space | $4.048\times10^{-12}$ |
| 4 | GO:CC | GO:0005576 | extracellular region | $3.436\times10^{-16}$ |
| 5 | GO:CC | GO:0031012 | extracellular matrix | $3.295\times10^{-18}$ |
| 6 | GO:CC | GO:0062023 | collagen-containing extracellular matrix | $9.763\times10^{-17}$ |
| 7 | GO:MF | GO:0005201 | extracellular matrix structural constituent | $2.717\times10^{-17}$ |

Supplementary Figure 10: The significant signalling pathways found from the top 100 genes for BRCA when breast normal tissue is used as the reference. Labels 1 to 7 are the most significant pathways and their pathway names listed in the table below, with their respective significance value.

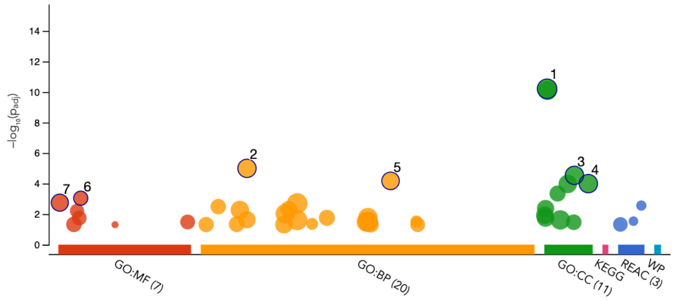


| ID | Source | Term ID | Term Name | $p_{adj}$ (query_1) |
|---|---|---|---|---|
| 1 | GO:CC | GO:0005576 | extracellular region | $6.193\times10^{-11}$ |
| 2 | GO:BP | GO:0009888 | tissue development | $1.005\times10^{-5}$ |
| 3 | GO:CC | GO:0070062 | extracellular exosome | $2.880\times10^{-5}$ |
| 4 | GO:CC | GO:1903561 | extracellular vesicle | $1.005\times10^{-4}$ |
| 5 | GO:BP | GO:0060429 | epithelium development | $6.663\times10^{-5}$ |
| 6 | GO:MF | GO:0008201 | heparin binding | $9.085\times10^{-4}$ |
| 7 | GO:MF | GO:0000981 | DNA-binding transcription factor activity, RNA ... | $1.779\times10^{-3}$ |

Supplementary Figure 11: A detailed view of the top signalling pathways for BRCA classification using a random training sample as the reference sample. There is a decrease in pathways involving the extracellular matrix compared to when normal breast tissue was used as a reference value.

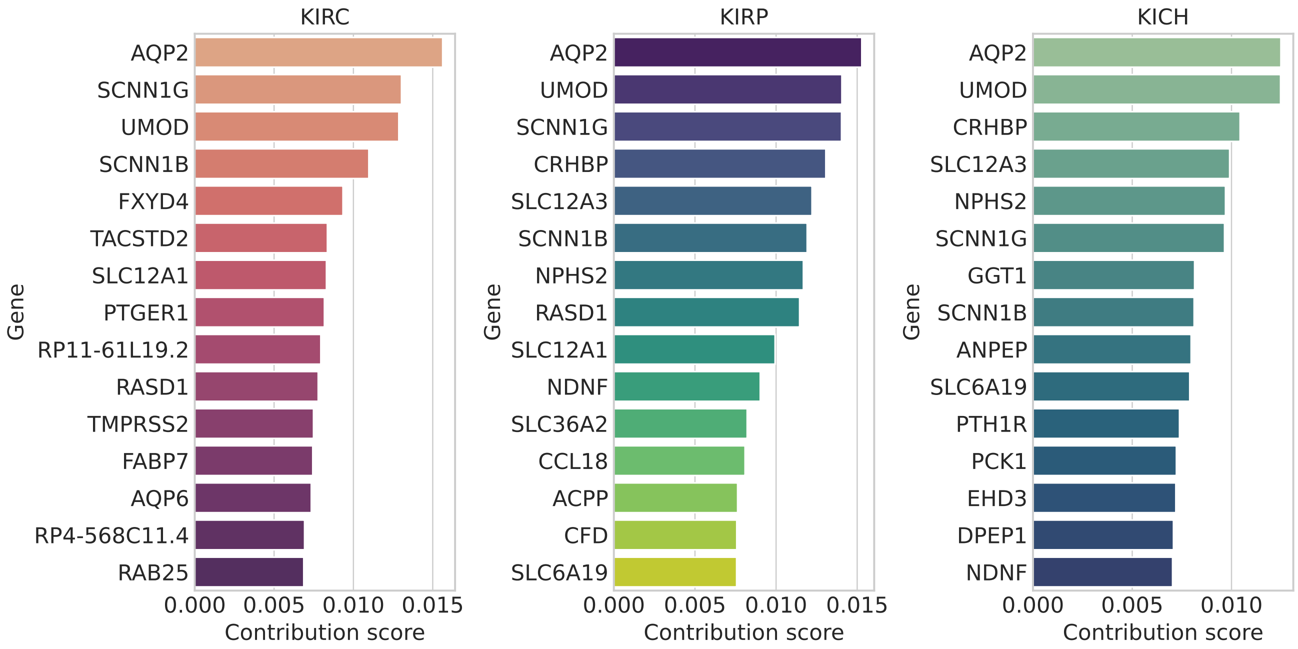


Supplementary Figure 12: The top 15 genes for the common dimension 35 among KICH, KIRC and KIRP. APQ2 is the most important gene in all three cases.
